# Amide chemical exchange saturation transfer at 7 T: a possible biomarker for detecting early response to neoadjuvant chemotherapy in breast cancer patients

**DOI:** 10.1186/s13058-018-0982-2

**Published:** 2018-06-14

**Authors:** Erwin Krikken, Vitaliy Khlebnikov, Moritz Zaiss, Rajni A. Jibodh, Paul J. van Diest, Peter R. Luijten, Dennis W. J. Klomp, Hanneke W. M. van Laarhoven, Jannie P. Wijnen

**Affiliations:** 1 Department of Radiology, University Medical Center Utrecht, Utrecht, The Netherlands; 2 Max Planck Institute for Biological Cybernetics, Tübingen, Germany; 3 Department of Medical Oncology, Academic Medical Centre Amsterdam, Amsterdam, The Netherlands; 4 Department of Pathology, University Medical Center Utrecht, Utrecht, The Netherlands

**Keywords:** 7 T MRI, APT CEST, NAC treatment, Breast cancer, Response prediction

## Abstract

**Background:**

The purpose of this work was to investigate noninvasive early detection of treatment response of breast cancer patients to neoadjuvant chemotherapy (NAC) using chemical exchange saturation transfer (CEST) measurements sensitive to amide proton transfer (APT) at 7 T.

**Methods:**

CEST images were acquired in 10 tumors of nine breast cancer patients treated with NAC. APT signals in the tumor, before and after the first cycle of NAC, were quantified using a three-pool Lorentzian fit of the z-spectra in the region of interest. The changes in APT were subsequently related to pathological response after surgery defined by the Miller-Payne system.

**Results:**

Significant differences (*P* <  0.05, unpaired Mann-Whitney test) were found in the APT signal before and after the first cycle of NAC in six out of 10 lesions, of which two showed a pathological complete response. Of the remaining four lesions, one showed a pathological complete response. No significant difference in changes of APT signal were found between the different pathological responses to NAC treatment (*P* > 0.05, Kruskal-Wallis test).

**Conclusions:**

This preliminary study shows the feasibility of using APT CEST magnetic resonance imaging as a noninvasive biomarker to assess the effect of NAC in an early stage of NAC treatment of breast cancer patients.

**Trial registration:**

Registration number, NL49333.041.14/NTR4980. Registered on 16 October 2014.

## Background

Neoadjuvant chemotherapy (NAC) is a systemic therapy that downstages cancer, enabling breast-conserving surgery and reducing axillary treatment [1–4]. Unfortunately, patients undergoing NAC may experience severe side effects and, in approximately 20% of patients, treatment does not result in tumor size reduction [5, 6]. To spare patients from ineffective treatment, it would be beneficial to predict the pathological response early in the course of treatment, allowing adjustments for patient-specific therapy.

Currently, the effect of NAC is generally evaluated based on change in the size of the tumor. Since underlying tumor changes in response to treatment usually precede a relatively slow process of change in tumor size [7–9], we set out to investigate metabolic properties of breast cancers that are expected to reveal changes early in the course of treatment.

Image contrast in standard clinical magnetic resonance imaging (MRI) is based on the concentration of water protons, and T_2_- and T_1_-weighted imaging reveal anatomical information of different tissues. A recently developed MRI method, chemical exchange saturation transfer (CEST), allows detection of interactions between metabolites and water in the body [10, 11].

Amide proton transfer (APT) CEST MRI detects the transfer of magnetization of labeled amide protons (resonating at 3.5 ppm downfield from the water) [12] and is sensitive to cellular mobile protein content as well as tissue pH. NAC treatment may cause an effect on these properties; therefore, we expect to observe changes in APT early in the course of treatment which can be predictive of the pathological response. Dula et al. also assessed the reproducibility of APT imaging in breast cancer patients at 3 T and showed group changes in APT during NAC, suggesting it may predict treatment response [13]. Going to higher field strength (7 T) will improve the signal to noise ratio (SNR) and contrast to noise ratio (CNR), improving the sensitivity of APT signals to therapeutic response [14]. Furthermore, endogenous T_1_ relaxation times become longer with increasing field strength, resulting in an increase in CEST signals [15–18].

A few studies have shown reproducible methods which maximize the SNR for APT CEST MRI in the breast at 7 T. They measured the APT effect with a standard deviation of 1% in healthy glandular tissue [14, 19]. Assuming approximately the same standard deviation in tumor tissue, in this preliminary study we set out to investigate the possibility of using personalized APT MRI at 7 T to evaluate the effect of NAC treatment in a cohort of nine breast cancer patients.

## Methods

### Subjects

This MRI study was performed in accordance with the guidelines of the UMC Utrecht ethics committee and was part of a larger study. Nine breast cancer patients with a total of 10 lesions gave informed consent to participate in this study. Patients being treated with NAC were selected, and they were examined with APT CEST MRI before (pre-) and after (post-) the first cycle of NAC (at approximately 3-week intervals). Table 1 summarizes their demographic and tumor characteristics.

**Table 1:** Patient demographics and tumor characteristics

Patient	Age (years)	Treatment regimes	ER	PR	HER2 neu	Diameter (mm)^a^	TNM stage
1	61	4×AC–12×paclitaxel	+	–	+	24	T2N1M0
2	50	3×FEC–3×docetaxel	+	+	–	30	T4N1M0
3R	57	3×FEC–3×docetaxel	+	+	–	35	T2N0M0
3L	57	3×FEC–3×docetaxel	+	+	–	15	T1N0M0
4	43	3×FEC–3×docetaxel	+	–	–	24	T2N0M0
5	59	3×FEC–3×docetaxel	+	+	–	32	T2N1M0
6	39	3×FEC–3×docetaxel	+	–	–	110	T3N2M0
7	55	6×Taxotere–AC	–	–	–	32	T2N0Mx
8	63	6×Taxotere–AC	+	+	–	15	T2N1Mx
9	35	6×Taxotere–AC	–	–	–	30	T2N0M0

*AC* adriamycin and cyclophosphamide, *ER* estrogen receptor, *FEC* 5-fluorouracil, epirubicin, and cyclophosphamide, *HER2* human epidermal growth factor receptor 2, *L* left, *PR* progesterone receptor, *R* right, *TNM* tumor, nodes, metastasis (classification of malignant tumors)

^a^ Measurement performed on 3 T magnetic resonance imaging acquired for standard clinical practice

### Acquisition

All patients were scanned in a prone position on a 7 T MR system (Philips, Cleveland, OH, USA). Six patients were scanned with a 26-channel bilateral breast ^1^H transceiver coil (MR Coils, Zaltbommel, The Netherlands) [20] and three patients were scanned with a 2-channel unilateral ^1^H/^31^P dual-tuned transceiver coil (MR Coils, Zaltbommel, The Netherlands) [21]. Two setups were used because this was a multicenter study and the RF coil was different in the two facilities. Third-order image-based B_0_ shimming was performed with least square error optimization using a 3D B_0_ map followed by manual segmentation of the breasts [22].

CEST-MRI was performed using a series of 20 sinc-Gauss radiofrequency (RF) pulses (pulse duration: 100 ms; inter pulse delay: 100 ms; the peak amplitude B_1_ ≈ 2 μT) resulting in a 4-s saturation train (50% duty cycle) followed by a gradient-echo readout [23]. Image acquisition included fat suppression with a short 1–2–1 spectral-spatial RF pulse to allow for a short TE of 1.4 ms, a TR of 2.6 ms, and a flip angle of 1.2 °. A coronal field of view (FOV) of 150 × 320 × 100 mm^3^ (FH × RL × AP) with a true resolution of 2.3 × 3.0 × 6.8 mm^3^ was obtained in two shots with an interval of 4.48 s and a fourfold acceleration in the right-left direction; 33 frequency offsets were acquired resulting in a scan time of 4 min 55 s. These offsets were not equally distributed over the frequencies; more offsets were obtained around the amide peak (3.5 ppm) and the water peak (0.0 ppm) for better fitting of these resonances. The frequency offsets associated with the nuclear Overhauser effect (NOE) were not included due to signal distortions by unsuppressed lipid resonances.

In the same MRI session, the last acquired scan was a dynamic contrast-enhanced (DCE) series. The first high-resolution scan after contrast administration was used to aid delineating the tumor on the CEST images. The dynamic series consisted of 18 consecutive 3D T_1_-weighted gradient echo sequences, starting with a fat-suppressed high-resolution scan prior to the contrast injection of 0.1 mmol/kg gadobutrol (Bayer Schering Pharma AG, Berlin, Germany), followed by 12 high-temporal resolution scans (TE = 1.6 ms, TR = 4.8 ms, flip angle = 8 °, FOV = 160 × 350 × 160 mm^3^, resolution = 2.86 × 2.86 × 2.86 mm^3^) to finish with another five fat-suppressed high-resolution scans (TE = 2.5 ms, TR = 5.6 ms, flip angle = 8 °, FOV = 160 × 350 × 160 mm^3^, resolution = 0.7 × 0.7 × 0.7 mm^3^).

### Data analysis

**Fig. 1:**
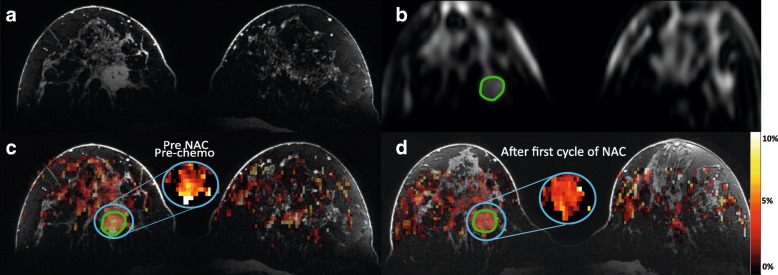
MR imaging dataset from patient 1 (see Table 1). **a** The last image of the DCE series was used to aid in drawing the ROI in the tumor. **b** S_0_ image (acquired at 33.6 ppm) was used to draw the tumor ROI (green circle) before applying this on the APT map. **c** Calculated APT map on top of the DCE image from the dataset before the patient started with neoadjuvant chemotherapy (NAC) treatment. **d** Calculated APT map after the first cycle of NAC treatment. The blue circles indicate the insert of the tumor region for better viewing of the underlying APT map. Color bar = percentage of APT effect

Image processing and data analysis were performed with MATLAB 2014b (MathWorks, Natick, MA, USA). CEST images were B_0_ corrected using the WASSR method [24]. A region of interest (ROI) was drawn in the whole tumor using the MR image of the last offset (33.6 ppm) of the CEST series before and after the first NAC. The procedure for selecting the ROI in the tumor can be seen in the upper part of Fig. 1 for one single slice. Figure 1a shows the DCE images which were used to aid in selecting the tumor region. The S_0_ image acquired at 33.6 ppm (Fig. 1b) was used to draw the ROI in the tumor (the green circle in Fig. 1b). To obtain a mean APT signal in the whole tumor, this procedure was performed for all the slices containing the tumor. The mean APT signal in the ROI, as a function of the frequency (z-spectra), was fitted with a three-pool Lorentzian model (water, APT, and magnetization transfer (MT)) using the Levenberg-Marquardt algorithm (see Table 2 for fit parameters [16, 25]):

1 $$ \frac{M_z\left(\Delta  \omega \right)}{M_z^0\left(\Delta  \omega \right)}=Z\left(\Delta  \omega \right)={Z}_{base}-{\sum}_i{L}_i\left(\Delta  \omega \right) $$

with

2 $$ {L}_i\left(\Delta  \omega \right)={A}_i\frac{\raisebox{1ex}{${\varGamma}_i^2$}\!\left/ \!\raisebox{-1ex}{$4$}\right.}{\raisebox{1ex}{${\varGamma}_i^2$}\!\left/ \!\raisebox{-1ex}{$4$}\right.+{\left(\Delta  \omega -{\delta}_i\right)}^2} $$

**Table 2:** Starting points and boundaries of all fit parameters of the three-pool Lorentzian fit.

	Start	Lower	Upper
Z_base_	0.5	0.5	1
A_water_	0.8	0	1
Γ_water_	1	0.1	2.5
δ_water_	0	–1	1
A_MT_	0.1	0	1
Γ_MT_	5	3	100
δ_MT_	0	−0.5	0.5
A_amide_	0.1	0	1
Γ_amide_	1	1	1.5
δ_amide_	3.5	3.3	3.7

The chemical shift δ and FWHM Γ are given in ppm

*Γ* FWHM, *δ* chemical shift, *MT* magnetization transfer

Each Lorentzian function L_i_ of effect *i* is defined for the offset frequency Δω by amplitude A_I_, full width at half maximum Γ_i_, and displacement from the frequency of free water protons δ_i_. The parameter Z_base_ corrects for constant signal reduction. The calculated APT map was obtained using the amplitude of the fit of the APT signal. To facilitate the comparison of different lesions in terms of APT signal change following the first NAC cycle, the mean APT signal pre-NAC was normalized to 1. The whole tumor analysis was compared to a single-slice approach containing the largest diameter of the tumor.

### Pathology

The pathological responses to NAC are defined according to the Miller-Payne system [26]; complete response is classified as grade 5 and nonresponse as grade 1. Pathological nonresponse indicates no change or some alteration to individual malignant cells, but no reduction in overall cellularity compared to pretherapy core biopsy. We grouped grades 1 and 2 together as nonresponders, grades 3 and 4 as partial responders, and grade 5 as complete responders.

### Statistical analysis

Statistical analysis was performed using an unpaired Mann-Whitney test (GraphPad Prism, GraphPad Software, San Diego, CA, USA), with a two-tailed distribution to show statistical difference (α = 0.05) between the APT signal pre-NAC and after the first cycle of NAC.

A Kruskal-Wallis test with a post-hoc Dunn’s multiple comparison test was used to assess statistical difference in APT signal between the groups with different pathological responses (nonresponders, partial responders, and complete responders).

## Results

### Data analysis

Figure 1c and d present calculated color APT maps manually overlaid on the image of the DCE series before (Fig. 1c) and after (Fig. 1d) the first cycle of NAC treatment. Within this tumor, a reduced amide signal of 21% in the APT map was observed after the first cycle of NAC.

**Fig. 2:**
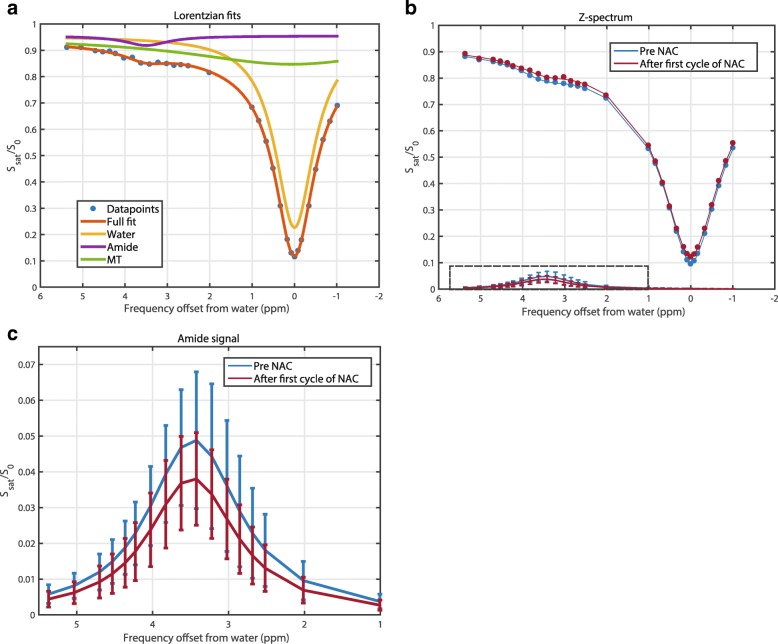
Results of the CEST analysis in the same patient as in Fig. 1 in the tumor ROI (see Table 1, patient 1). **a** Results of the three-pool Lorentzian fitting of the Z-spectrum of water (yellow line), magnetization transfer effect (MT; green line), amide proton transfer (APT; purple line), and the full fit consisting of the three fits (orange). **b** Mean Z-spectrum in the tumor before (blue) and after (red) the first cycle of neoadjuvant chemotherapy (NAC) treatment. The insert of the dashed box is shown in **c**, which shows the Lorentzian fit for the APT signal before (blue) and after (red) NAC treatment with the standard deviation of the APT signal of the voxels in the tumor ROIs, shown as the error bars

An exemplary three-pool Lorentzian fit is depicted in Fig. 2a. The acquired data points from the tumor ROI (green circle in Fig. 1c) are shown as blue dots representing the Z-spectrum magnitude at the 32 frequency offsets, the water fit as a yellow line, the MT fit as a green line, the APT fit as a purple line, and the full fit consisting of the three fits in orange. Figure 2b shows the corresponding Z-spectrum within the tumor before (blue line) and after the first cycle of NAC treatment (red line) based on the 32 frequency offsets from the same case as in Fig. 1. Figure 2c is the insert of the dashed box in Fig. 2b, presenting the Lorentzian fit for the APT signal before (blue) and after (red) NAC treatment. The 21% decrease in APT signal after the first cycle of NAC treatment can be observed.

**Fig. 3:**
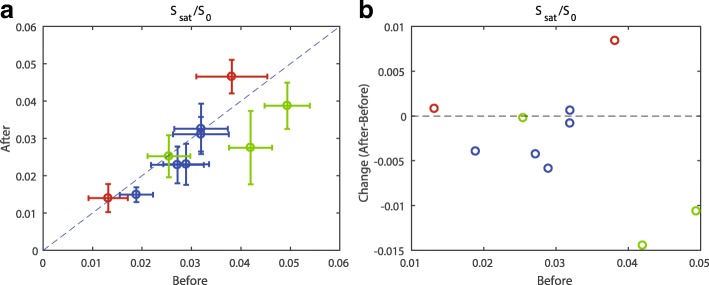
Mean APT signal and standard deviation within the ROI (tumor) for all ten lesions. **a** The mean APT signal after the first cycle of NAC is plotted versus the mean APT signal before the start of NAC for the nonresponders (red), partial responders (blue), and complete responders (green). **b** The mean change in APT signal is plotted versus the mean APT signal before NAC. The dashed lines in both **a** and **b** indicate the absence of a change in APT signal before and after the first cycle of NAC treatment

The mean and standard deviation of the APT signal inside the tumor before and after the first cycle of NAC for all the ten lesions, together with the pathological response, are shown in Table 3. The asterisks in Table 3 indicate a significant difference comparing the APT signal before and after the first cycle of NAC treatment of each patient. Table 3 is further visualized in Fig. 3 where the nonresponders are shown in red, the partial responders in blue, and the complete responders in green. The dashed lines in Fig. 3 indicate the absence of a change in the APT signal before and after the first cycle of NAC treatment.

**Table 3:** Mean amide proton transfer (APT) signal in the tumor before and after the first cycle of neoadjuvant chemotherapy (NAC)

Patient	Pathological response	Mean (± SD) APT signal before NAC	Mean (± SD) APT signal after the first cycle of NAC	Delta mean APT signal	*P* value^a^
1	5	0.0494 (± 0.0062)	0.0388 (± 0.0046)	− 0.0106	< 0.0001*
2	3	0.0319 (± 0.0046)	0.0311 (± 0.0056)	−0.0008	0.0458*
3R	4	0.0289 (± 0.0055)	0.0231 (± 0.0046)	−0.0058	0.0004*
3L	4	0.0189 (± 0.0020)	0.0149 (± 0.0034)	−0.0040	0.0003*
4	5	0.0420 (± 0.0098)	0.0275 (± 0.0043)	−0.0145	< 0.0001*
5	2	0.0132 (± 0.0037)	0.0140 (± 0.0040)	+0.0008	0.5749
6	4	0.0272 (± 0.0049)	0.0229 (± 0.0053)	−0.0043	0.4135
7	5	0.0254 (± 0.0056)	0.0252 (± 0.0043)	−0.0002	0.5259
8	3	0.0319 (± 0.0068)	0.0326 (± 0.0054)	+0.0007	0.4170
9	2	0.0381 (± 0.0045)	0.0465 (± 0.0072)	+0.0084	0.0001*

^a^Unpaired Mann-Whitney test with two-tailed distribution

*Significant difference

**Fig. 4:**
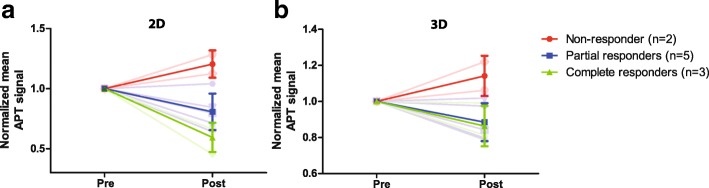
Normalized changes in amide proton transfer (APT) signal after the first cycle of neoadjuvant chemotherapy in the slice with the largest tumor diameter **a** and in the whole tumor **b**. The mean of the nonresponders (bright red line), partial responders (bright blue line), and complete responders (bright green line) on top of the change in APT signal of all the lesions (transparent lines in the background), where each line represents a different lesion. The standard deviation in each group is shown as error bars

Normalized changes in APT signal after the first cycle of chemotherapy with pre-NAC APT signal normalized to 1 for all the tumors can be observed in Fig. 4. The one-slice approach (slice with largest tumor diameter; Fig. 4a) was compared with the change in the APT signal in the whole tumor (Fig. 4b). A distinction between the mean of the nonresponders (bright red line), the mean of the partial responders (bright blue line), and the mean of the complete responders (bright green line) is visible. Each blurred line in the backgrounds represents a different patient and the standard deviation in each group is shown as error bars. Note that the distinction between the partial responders and complete responders in the single-slice approach is clearer than in the change of APT signal in the whole tumor.

### Statistical analysis

Based on the 3D whole-tumor analysis, six out of the ten lesions showed a significant change in the APT signal in the tumor, where two out of three complete responders (patients 1 and 4) showed a significant difference (*P* <  0.0001 for both patients; Table 3). The third complete responder, patient 7, showed no significant difference (*P* = 0.5259). Of the partial responders, three lesions (patients 2 and 3R and 3L) also showed a significant difference in APT signal (*P* = 0.0458, *P* = 0.0004, and *P* = 0.0003, respectively), whereas the other partial responders (patients 6 and 8) showed no significant differences (*P* = 0.4135 and *P* = 0.4170, respectively). One nonresponder showed a significant difference (patient 9, *P* <  0.0001) while the other nonresponder showed no statistical difference between the APT signal before and after the first cycle of NAC (patient 5, *P* = 0.5749). However, note that both nonresponders showed an increase in APT signal while the other responders (patient 8 as an exception) showed a decrease in the APT signal, suggesting that the nonresponders can be differentiated quite well from the responders based on the direction of the change in the APT signal.

The Kruskal-Wallis test with the post-hoc Dunn’s multiple comparison test revealed no significant difference in the change of APT signal after the first cycle of NAC (*P* = 0.1057) between the three different pathological responses.

## Discussion

In this preliminary study, the potential for using CEST at 7 T to monitor treatment effects in breast cancer was evaluated. We demonstrated that amide CEST signals can be measured in breast cancer before and after the first cycle of NAC. Significant differences in the APT CEST MRI signal were observed (*P* <  0.05) in six out of ten breast tumors. For the direction of the change in APT signal, the nonresponders (patients 5 and 9) showed an increase in APT signal whereas the other patients (except patient 8) showed a decrease in the APT signal. A larger patient population is required for a more comprehensive analysis of change in APT signal in relation to pathological response.

Animal studies [27, 12] demonstrated increased APT effects in tumors compared with healthy tissue, and Salhotra et al. attributed this effect to increased cellular proliferation and subsequent accumulation of defective proteins [27, 28]. In breast tumors, Dula et al. [13] observed changes in the APT signal and attributed this to changes in the concentration of proteins and peptides or in the amide proton exchange rates, influenced by a change in pH, and perhaps T_1_ [12, 29–33] due to therapy effects. Remarkably, the nonresponders in this preliminary study showed an increase in APT signal after the first cycle of NAC, which possibly hints at a continuing increase in the concentrations of proteins and peptides since the tumor was not affected by the NAC treatment. However, the increase in APT signal in the tumor is likely related to a combination of all the abovementioned effects. Assuming these processes occur at the core of the tumor, the largest expected change in the APT signal would be at that location. This could explain the larger APT increase observed in the single-slice (with largest tumor diameter) analysis compared to the 3D whole-tumor volume analysis. (Fig. 4). As for the single-slice approach, the core contributes relatively more to the APT signal resulting in a larger mean change in APT signal compared with the mean change in APT signal of the whole tumor. It also seems that in the single-slice approach the distinction between the partial responders and the complete responders becomes clearer, hinting at a more linear distribution of change in APT signal for the three response groups. Further research needs to be performed to examine if the single-slice approach results in a better distinction between the different response groups. Although a single-slice approach could save scan time or allow for more frequency offsets in the same scan time, caution is needed as it may be prone to unwanted interinstitutional variations due to differences in slice selection.

Although patient 7 was a complete responder, the tumor APT signal change (Fig. 3) was located in the middle of the partial responders (blue), suggesting the same change in APT signal as the partial responders. Interestingly, this was the only triple negative tumor from the three complete responders. Triple negative tumors feature a unique microenvironment distinct from that of other subtypes [34, 35], possibly explaining the different APT signal in this type of tumor; however, this observation remains to be confirmed.

Due to the small sample size (*n* = 10) we did not correct for age or menstrual cycle effects, which are known to affect the breast density. The water content of the breast parenchyma particularly changes during the menstrual cycle [36], possibly influencing the CEST effect [16, 17]. Additionally, all patients in this study received NAC treatment which is known to influence the menstrual cycle.

The overall calculated APT contrast can also be influenced by the degree of fat suppression. We used RF and gradient spoiling to reduce lipid artefacts. However, insufficient fat suppression in the tumor may have resulted in an underestimation of the CEST amplitude [37], possibly affecting the change in APT signal.

CEST MRI is sensitive to the B_1_^+^ field, since different exchanging groups are affected by the B_1_^+^. Variation in B_1_^+^ may cause more contrast from slower (or faster) exchanging species. We were interested in the APT CEST MRI signal generated by the slow exchanging amide protons, and the optimal B_1_^+^ for detecting this exchange is approximately 1 μT [38]. The peak B_1_ amplitude in this study was set to 2 μT to account for B_1_ loss due to the hardware setup. The unilateral setup consisted of a quadrature RF coil placed in front of the breast, while the bilateral setup was an exact copy of this coil, only for both breasts. This setup led to a decrease in B_1_, varying from 60% in the front of the breast to 50% towards the pectoralis major. The sequence was optimized to achieve an acquired B_1_ of approximately 1 μT throughout the breast. Therefore, the location of the tumor can possibly influence the measured change in the APT CEST signal. In this study, we assumed that the location of the tumor was the same between the two measurements receiving the same amount of B_1_ and therefore not influencing the measured change in the APT CEST signal. Several B_1_^+^ inhomogeneity correction methods have been proposed [25, 39, 40] to improve the CEST images. These methods, however, require an accurate B_1_ map or acquisition at multiple B_1_ amplitudes. Due to limited scan time this was unfortunately not possible in this study. Another solution to deal with B_1_ inhomogeneity would be a new design for the coil setup at 7 T where a homogeneous B_1_ field can be achieved. For example, the use of dipole antennas has been shown to achieve this in body imaging [41]. These methods for improving the B_1_ field could be good options for future studies to further improve the quality of the APT maps of the breast.

It would be interesting to translate these findings to the clinical field strength of 3 T. Klomp et al. [14], however, compared APT CEST MRI at 3 T with 7 T and found that the variance of noise in measurements at 3 T was large (i.e., approximately 5% of CEST effects when compared with the water signal, larger than the alterations in CEST effects we have observed in our study), thereby limiting the ability to discern subtle changes during, for example, first-line therapy. At 7 T, however, the increased SNR and increased spectral resolution enabled a fourfold reduction in the noise of the observed APT CEST compared with 3 T. Due to the inherently small CEST signal, at the moment changes within these CEST effects seem challenging to detect at 3 T. Fortunately, 7 T has been Food and Drug Administration (FDA) approved and thereby will likely become available to more hospitals.

Predictive biomarkers are critical for the evaluation of neoadjuvant therapies, as well as the effect of novel targeted agents intended to be incorporated into neoadjuvant therapy [42]. The potential for high-field-strength CEST MRI of the breast as a biomarker for the evaluation of NAC response is promising, as is the capability to noninvasively detect changes in protein and peptide levels. These may play a key role in understanding breast tumor progression and response to treatment [19].

## Conclusions

In a small group of breast cancer patients, this preliminary study shows the feasibility of using APT CEST MRI at 7 T as a noninvasive biomarker to predict the effect of neoadjuvant chemotherapy in an early stage of treatment. This can be useful in personalized breast cancer treatment planning.

## Abbreviations

APT: Amide proton transfer
CEST: Chemical exchange saturation transfer
CNR: Contrast to noise ratio
DCE: Dynamic contrast-enhanced
MRI: Magnetic resonance imaging
MT: Magnetization transfer
NAC: Neoadjuvant chemotherapy
NOE: Nuclear Overhauser effect
RF: Radiofrequency
ROI: Region of interest
SNR: Signal to noise ratio
T: Tesla
